# Mr. Richard Phillips' Translation of the New Pharmacopœia, with Notes, &c.

**Published:** 1825-04-01

**Authors:** 


*A Translation of the Pharmacopœia of the Royal College of Physicians of London*, 1824, *with Notes and Illustrations*. By Richard Phillips, F.R.S. L. et E. &c., Lecturer on Chemistry, &c. Octavo, pp. 326, price 8s. 6d. boards. London, 1824.

Of *this* work, or of *this* translation of the work, it is not necessary to say any thing. The Pharmacopœia is now well known, and the translator's ability to perform this task is unquestionable. The notes and illustrations are all that we have any thing to do with. After what happened on a former occasion, the chemical and pharmaceutical world was in expectation of a tart critique from Mr. Phillips. But TIME seems to have rubbed off his critical asperities, and done what LETTERS are said to do.

> "Emollit mores, nec sinit esse feros."

Perhaps the work is not the less useful in consequence of this milder tone in its composition. Mr. Phillips's views in the accompanying notes, have been chiefly directed to what he conceived to be the wants of the medical student—"with the intention of offering him a concise explanation of the chemical changes which occur during the preparation of medicines—of the qualities which indicate their perfection—and of the means by which he may detect the adulterations to which they are subjected." But, although our author's attention has been principally bestowed on the *learner*, yet he flatters himself "that some of his experimental researches may prove not totally devoid of interest even to those who may long have been practitioners in medicine." He here alludes more particularly to the preparations of iron, especially to the nature and quantity of oxide which they contain, both when prepared according to the College, and as usually met with in the shops. He trusts his analyses and observations will be useful in causing greater uniformity in the preparations, and in guiding the practitioner to the selection of those which merit the greatest reliance. By the aid of diagrams, our author has facilitated the comprehension of those changes which take place in the compositions and decompositions of medicinal substances. He has also paid some attention to what he considers a curious and interesting, though much neglected, department of science—"the crystalline forms of salts." Not being a medical practitioner himself, he has drawn what is said of the powers and doses of medicines from the best sources—especially Dr. Paris's Pharmacologia and Mr. Thomson's Dispensatory.

Having thus pourtrayed the objects and intentions of our author, it only remains for us to exhibit some specimens of the execution, taken without much selection from the notes.

*Example* I. ANTIMONIUM TARTARIZATUM. After describing the process for preparing this important medicine, as directed by the College, and commending the improvement introduced into the present Pharmacopœia, our author goes on to remark thus:—

"The crystals of this salt are colourless and inodorous, but have a styptic metallic taste: on exposure to the air, they effloresce slightly, and become opaque. When heated with carbonaceous matter this salt is decomposed, and metallic antimony is obtained. It is soluble in about fifteen times its weight of water at 60°, and twice its weight at 212°. The aqueous solution decomposes spontaneously after it has been some time prepared. It is insoluble in alkohol.

*Composition.*—This is a double salt, or a compound of tartrate of potash and tartrate of antimony; but I am not satisfied with the results of any analysis hitherto given—that which is generally quoted, is obviously incorrect.

"*Adulteration.*—This salt should never be purchased in powder, but always in crystals: in the former state it frequently contains a portion of supertartrate of potash uncombined with any oxide, and which, in preparing the liquor antimonii tartarizati, is precipitated. To judge if the crystals have been properly prepared, drop one or two into a solution of sulphuretted hydrogen gas, and an orange-coloured deposite will be formed on them.

"*Incompatibles.*—The solution of this salt is decomposed by acids, by alkalies and their carbonates, by some of the earths and metals, and their oxides, by lime-water, muriate of lime, and the acetates of lead. Many vegetable infusions, and especially those which are bitter and astringent, decompose it, such as cinchona, rhubarb, catechu, &c." 83.

Speaking of the vinum antimonii tart: as prepared with dilute spirit, instead of wine, he observes that—"when tartarized antimony has been carelessly prepared, and contains supertartrate of potash uncombined with oxide of antimony, it is precipitated from solution in water by spirit; those practitioners, therefore, who purchase tartarized antimony, should insist on having it in the state of crystals, in which there is little chance of the occurrence of this imperfection."

On analysing specimens of PULVIS ANTIMONIALIS, procured from two respectable sources, he found one to consist of 35 parts of peroxide of antimony and 65 of phosphate of lime. The other specimen shewed 38 parts of the peroxide to 62 of the phosphate. Mr. Phillips also analysed James's powder, and found it to consist of nearly the same ingredients and proportions. He concludes, that both forms are perfectly inert. In this we do not agree with our author, simply because we have seen, times innumerable, sickness and vomiting produced by both preparations.

*Ex.* 2. *Ferri Subcarbonas.*

" *Composition and Qualities.*—This preparation is usually of a deep red colour; and it consists generally of about

| | |
|---|---|
| Carbonate of iron . . . . . . | 4 |
| Peroxide of iron . . . . . . | 96 |
| | 100 |

" I have procured this preparation from several respectable sources; but have never found it to contain five per cent. of carbonate of iron: the reason of this is, that unless it be washed with hot water, and then speedily dried, the protoxide of iron absorbs oxygen, and being converted into peroxide, it is no longer capable of remaining combined with carbonic acid; for a solid percarbonate of iron cannot be formed, though it may probably exist in solution.

" When preparing this compound with the greatest care, I have found it to contain about 15 per cent. of carbonic acid, which indicates its composition to be

| | |
|---|---|
| Carbonate of iron . . . . . . | 40 |
| Peroxide of iron . . . . . . | 60 |
| | 100 |

" From the great affinity which exists between iron and oxygen, and from the rapidity with which the protoxide absorbs it, so as to form peroxide, it seems to be impossible to procure a perfect carbonate or subcarbonate of iron.

" Ferri subcarbonas, when it has been carefully made, has a reddish-brown colour; it dissolves readily in acids with effervescence, and yields 15 per cent. of carbonic acid during solution. When improperly prepared it contains only about 1.5 per cent. of carbonic acid, its colour is less brown, and it is not so easily dissolved by acids.

" When this preparation contains a large proportion of carbonic acid, it is much more readily soluble, and its activity as a medicine is probably proportional to the quantity of that acid present.

" *Incompatibles.*—Acids and acidulous salts, which dissolve it with effervescence." 98.

We have only room for one more example, and that from the same metal—iron.

### *Ex.* 3. *Tinct. Ferri Muriatis.*

"*Process.*—The muriatic acid decomposes the carbonate of iron, expelling the carbonic acid gas with effervescence, dissolving the protoxide of iron with which it was combined, and also the peroxide of iron. I find that the proportions here directed answer extremely well, for less than one scruple of the subcarbonate of iron remains undissolved, including the accidental impurity which it may contain.

"When this tincture is made with subcarbonate of iron, containing 15 per cent. of carbonic acid, it is a mixed solution of protomuriate and permuriate of iron in spirit; and its preparation from carbonate of iron is attended with this inconvenience, that the protoxide of iron becomes peroxide by absorbing oxygen, and part of it is precipitated, which renders the tincture liable to diminish and vary in strength.

"As Tinctura ferri muriatis is esteemed to be one of the most active of all the preparations of iron, I have paid particular attention to the preparation of it, and have procured it from several respectable sources for the purpose of examining the proportion of iron which it contains.

"The specific gravity of four different samples, and the proportions of peroxide of iron obtained from half a fluid ounce of each, were as follows:

| | | | |
|---|---|---|---|
| No. 1, | sp. gr. 1·030 | Peroxide of iron | 20· grains |
| 2, | 0·975 | | 11·3 |
| 3, | 0·951 | | 9·3 |
| 4, | 1·003 | | 12·1 |

"Finding these great variations in the specific gravities, and the quantities of oxide contained in these samples, I prepared some of the tincture precisely according to the directions of the College, and found its sp. gr. and the peroxide of iron yielded by half a fluidounce, were as follows:

Specific gravity 0·994 Peroxide of iron 16·8 grains.

"It is difficult to account for the excess of iron in the sample No. 1, but the deficiency of the remainder is readily explained by supposing, that the muriatic acid employed was too weak to dissolve the assigned portion of the subcarbonate.

"*Qualities and Composition.*—This tincture is of a reddish-brown colour, its taste is extremely styptic, and its odour resembles that of muriatic ether. When prepared, as it usually is, from peroxide of iron nearly unmixed and carbonate, it is almost entirely permuriate of iron, and each fluidrachm contains nearly 4·2 grains of peroxide of iron.

"*Incompatibles.*—Alkalies and their carbonates, lime-water, carbonate of lime, magnesia and its carbonate. It is rendered black by astringent vegetable bodies, and is decomposed by a solution of gum arabic." 100.

Upon the whole, we consider our author's notes and illustrations as very valuable additions to the Pharmacopœia, with very trifling increase of price.

